# Supplementary material for: Time to elective surgery and its predictors after first cancellation at Debremarkos Comprehensive Specialized Hospital, Northwest Ethiopia
Source: PLOS Glob Public Health. 2024 Jan 17;4(1):e0002183. doi: 10.1371/journal.pgph.0002183 (PMC10793890; doi:10.1371/journal.pgph.0002183)
Supplement: Attachment — Submitted filename: PGPH-D-23-00985_reviewer.pdf [file pgph.0002183.s002.pdf]

PLOS Global Public Health

Time to Surgery after First Cancellation and Its Predictors in Elective Surgery Cases at Debremarkos Comprehensive Specialized Hospital, Northwestern Ethiopia: Time to Event Analysis

--Manuscript Draft--

Manuscript Number:	PGPH-D-23-00985
Article Type:	Research Article
Full Title:	Time to Surgery after First Cancellation and Its Predictors in Elective Surgery Cases at Debremarkos Comprehensive Specialized Hospital, Northwestern Ethiopia: Time to Event Analysis
Short Title:	Time to Event Analysis of Surgery Timing Following First Cancellation
Corresponding Author:	Melkamu Siferih Zeleke, MD<br>DMU: Debre Markos University<br>Debremarkos, Amhara region ETHIOPIA
Order of Authors:	Yibeltal Abiyu, MPH
	Zewudie Aderaw, MPH,PhD
	Lieltework Yismaw, MSC
	Mulatu Mengaw, MPH
	Getamesay Demelash, MSC
	Melkamu Siferih Zeleke, MD
Keywords:	predictors; time to surgery; first cancellation; elective surgery; Ethiopia
Abstract:	Background: Long waiting times for surgery is a long-standing common problem worldwide. Few studies have evaluated predictors of time to surgery for elective surgery cases after the first cancellation in the country, Ethiopia; and no similar study in our current study settings. Thus, the current study aimed to identify predictors of time to surgery after the first cancellation in elective surgery cases at Debremarkos Comprehensive Specialized Hospital.<br>Methods: Institution-based retrospective follow-up study was conducted on 386 elective surgery cases who were admitted to Debre Markos Comprehensive Specialized Hospital in Northwestern Ethiopia after the first cancellation from September 1, 2017, to August 31, 2022. Data were extracted from both the surgery cancellation registration log book and patient charts using a checklist. Patient charts were selected using a systematic random sampling technique. Data were entered into Epi-Data version 3.1 and then exported to STATA software version 14.1 for analysis. Multi-variable Cox proportional hazard regression was used to identify predictors of time to surgery at P<0.05. The association was summarized using an adjusted hazard ratio (AHR).<br>Result: The mean age of the participants was 41.01 + 18.61 years old (range: 1-85 years). There were 323 (83.6%) [95% CI: 79.6, 87.05] surgeries done after the first cancellation. The total person-days of observation were 10013 or 3 out of 100 elective major surgeries per day observation after the first cancellation. The median survival time was 25 (IQR: 17-40 days). Urban residence (AHR=1.62; 95% CI 1.26, 1.96), being a member of health insurance schemes (AHR=1.55; 95% CI, 1.24, 1.96), stable medical condition (AHR=1.43; 95% CI 1.13, 1.79) and performing ordered laboratory investigations (AHR 1.62; 95% CI, 1.29, 2.04) were significant predictors for time to elective surgery after first cancellation.<br>Conclusion: The in-patient surgery rate after the first cancellation was 83.6% despite many patients requiring surgery, but comparable to many previous findings. The median survival time to surgery after the first cancellation was 25 days. Health insurance coverage, residence in an urban area, stable health status, and status of ordered laboratory tests were predictive factors for the timing of surgery after the first cancellation. Clinicians must focus on timely ordering and determinations of investigations, careful monitoring of patients, facilitating health insurance coverage, and treating other comorbid medical conditions. Researchers should concentrate on

Powered by Editorial Manager® and ProduXion Manager® from Aries Systems Corporation

	robust study designs such as prospective cohort studies.
**Additional Information:**	
**Question**	**Response**
**Financial Disclosure**<br><br>Enter a financial disclosure statement that describes the sources of funding for the work included in this submission and the role the funder(s) played. This includes grants and any commercial funding of the work or authors.<br><br>This statement will be typeset if the manuscript is accepted for publication.<br><br>*Please review the submission guidelines and the instructions link below for detailed requirements and guidance.*	no funding agent
**Competing Interests**<br><br>On behalf of all authors, disclose any competing interests that could be perceived to bias this work.<br><br>This statement will be typeset if the manuscript is accepted for publication.<br><br>*Please review the instructions link below and PLOS Global Public Health's competing interests policy to determine what information must be disclosed at submission.*	no competing interests
**Data Availability**<br><br>Before publication, Authors are required to make fully available and without restriction all data underlying their findings. Please see our PLOS Data Policy page for detailed information on this policy.	The Stata data and checklist are available fro the main author upon reasonable request

A **Data Availability Statement**, detailing where the data can be accessed, is required at first submission. Insert your Data Availability Statement in the box below.<br><br>Please see the data reporting section of our submission guidelines for instructions on what you need to include in your Data Availability Statement.<br><br>This statement will be typeset if the manuscript is accepted for publication.<br><br>*PLOS allows rare exemptions to address legal and ethical concerns. If you have legal or ethical restrictions, please use the box below to detail these in full sentences for the Journal team to consider.*	

Powered by Editorial Manager® and ProduXion Manager® from Aries Systems Corporation

Manuscript

Click here to access/download;Manuscript;Yibeltal manuscript time to surgery plos format.edited.docx

Time to Surgery after First Cancellation and Its Predictors in Elective Surgery Cases at Debremarkos Comprehensive Specialized Hospital, Northwestern Ethiopia: Time to Event Analysis

Yibeltal Abiyu[1], Zewudie Aderaw[2], Lieltework Yismaw[3], Mulatu Mengaw[4], Getamesay Demelash[5], Melkamu Siferih[6]*

[1,4]Department of Public Health, College of Medicine and Health Sciences, Debremarkos University, Debremarkos, Ethiopia;[2]Department of Public Health, Saint Paul's Hospital Millennium Medical College, Ethiopia;[3]Department of Biostatistics, College of Computational Sciences, Debremarkos University, Debremarkos, Ethiopia; [5]Department of Anesthesia, College of Medicine and Health Sciences, Debremarkos University, Debremarkos, Ethiopia; [6]Department of Obstetrics and Gynecology, School of Medicine, Debremarkos University, Debremarkos, Ethiopia

*__Correspondence to: Melkamu Siferih: siferihmelkamu@gmail.com__

Email Address

YA: yibabiyu@gmail.com

ZA: zewdie1984@gmail.com

LY: lielt.yismaw@gmail.com

MM:mulatumengaw@gmail.com

GD:getademelash123@gmail.com

MS:siferihmelkamu@gmail.com

ABSTRACT

Background: Long waiting times for surgery is a long-standing common problem worldwide. Few studies have evaluated predictors of time to surgery for elective surgery cases after the first cancellation in the country, Ethiopia; and no similar study in our current study settings. Thus, the current study aimed to identify predictors of time to surgery after the first cancellation in elective surgery cases at Debremarkos Comprehensive Specialized Hospital.

Methods: Institution-based retrospective follow-up study was conducted on 386 elective surgery cases who were admitted to Debre Markos Comprehensive Specialized Hospital in Northwestern Ethiopia after the first cancellation from September 1, 2017, to August 31, 2022. Data were extracted from both the surgery cancellation registration log book and patient charts using a checklist. Patient charts were selected using a systematic random sampling technique. Data were entered into Epi-Data version 3.1 and then exported to STATA software version 14.1 for analysis. Multi-variable Cox proportional hazard regression was used to identify predictors of time to surgery at $P \leq 0.05$. The association was summarized using an adjusted hazard ratio (AHR).

Result: The mean age of the participants was 41.01 + 18.61 years old (range: 1-85 years). There were 323 (83.6%) [95% CI: 79.6, 87.05] surgeries done after the first cancellation. The total person-days of observation were 10013 or 3 out of 100 elective major surgeries per day observation after the first cancellation. The median survival time was 25 (IQR: 17-40 days). Urban residence (AHR=1.62; 95% CI 1.26, 1.96), being a member of health insurance schemes (AHR=1.55; 95% CI, 1.24, 1.96), stable medical condition (AHR=1.43; 95% CI 1.13, 1.79) and performing ordered laboratory investigations (AHR 1.62; 95% CI, 1.29, 2.04) were significant predictors for time to elective surgery after first cancellation.

Conclusion: The in-patient surgery rate after the first cancellation was 83.6% despite many patients requiring surgery, but comparable to many previous findings. The median survival time to surgery after the first cancellation was 25 days. Health insurance coverage, residence in an urban area, stable health status, and status of ordered laboratory tests were predictive factors for the timing of surgery after the first cancellation. Clinicians must focus on timely ordering and determinations of investigations, careful monitoring of patients, facilitating health insurance coverage, and treating other comorbid medical conditions. Researchers should concentrate on robust study designs such as prospective cohort studies.

Keywords: predictors, time to surgery, first cancellation, elective surgery, Ethiopia

INTRODUCTION

Elective surgery is defined as elective non-emergency surgery that is medically necessary but can be delayed for at least 24 hours (1). Although these surgeries are optional, they are often important and potentially life-changing, and some are same-day surgeries that do not require a hospital **(2, 3)** Worldwide, 3.5% of surgical procedure is performed by patients who need elective surgery, and one-third of the procedure is undertaken after the patient experience at least one cancelation **(1, 4).** Because this is a global problem, 29% of elective surgeries were completed after patients experienced at least one cancellation, with an overall average waiting time of 21 days, the WHO report found **(5)**.

Elective surgery after the 1st cancellation refers to surgery performed after the patient cancels once on the scheduled date of surgery**(6)**. Long waiting times for surgery have long been a common problem worldwide **(6, 7)**. After the first cancellation, the burden of waiting before surgery was only 15% in Europe and 7% in Africa **(8).** In developing countries, including Ethiopia, wait times for surgery after the first cancellation are common, which also increases the risk of death in hospitals **(9).** More than 72% of reasons to wait long for surgery after cancellation can be avoided **(10).** If the waiting time is long, the operating room and time cannot be used properly, causing inconvenience to patients and their families and causing psychological trauma to both patients and their families**(11, 12).**

Previously, time-to-surgery among select cases on the African continent, particularly Ethiopia, focused primarily on time from eligibility to surgery (4). Time to surgery is an excellent measure of the quality of hospital surgical management **(13).** Unlike Western countries, few studies have evaluated time to surgery and its predictors among elective cases after first cancellation in the country, Ethiopia; even we could not find a similar study in our current study settings.

Our observations showed that many patients suffer from severe pain, additional costs, emotional trauma, and feelings of hopelessness, and eventually, the patient may die if the operation is canceled again on the scheduled date.

Better assessment and understanding of time to surgery and predictors after the first due date cancellation will reduce subsequent complications in cases, re-surgery, and over-hospitalization, and improve the quality of care for patients who have undergone elective surgery.

So, this study was designed to evaluate time to surgery and its predictors among elective surgery cases after the first cancellation in Debre Markos Comprehensive Specialized Hospital, Northwestern Ethiopia.

This study will help clinicians and other service providers design interventions to reduce surgical time in hospitals by removing predictable factors that could be avoided.

METHODS and MATERIALS

Study Setting, Design, and Populations

A Five-year hospital-based retrospective follow-up study was conducted from September 1/2017 to August 31/2022 at Debre Markos Comprehensive Specialized Hospital. The data were collected from September 10, 2022, to September 30, 2022.

The hospital is found in Debre Markos town. It is located 295 km away from Addis Ababa, the capital city of Ethiopia, and 265 km from Bahir Dar the capital city of Amhara regional state. The hospital has 216 admission beds, 4 major operation tables, and 20 senior specialists who perform elective operations, 232 staff nurses, 57 staff physicians, 10 anesthetists, 197 other health professionals, 139 other supportive staff. The hospital is providing 24-hour service in a day. Debre Markos Comprehensive Specialized Hospital serves more than 3.5 million people in its catchment area and more than 1422 annual elective surgical cases were done, and more than 280 elective cases were canceled every year based on the annual reports of the hospital **(14)**.

All patients who were admitted for elective surgery but not done with the first intended day of surgery (DOS) in Debre Markos Comprehensive Specialized Hospital were source populations. All patients who were scheduled for elective surgery and canceled with the first intended day of surgery in the Departments of General Surgery, gynecology, obstetrics, and Orthopedics from September 1/2017 to August 31/2022 were studied populations. Elective surgery cases without documented important variables such as cancellation date and surgery date were excluded. In addition, minor surgery cases were excluded.

Sample Size Determination and Sampling Procedure

The sample size was calculated by using the sample size determination formula for survival analysis. It is calculated by taking the two-sided significant level of (α) of 5%, power 80%

Sample size (n) = $\frac{\text{number of events}}{\text{Probability of event}}$, number of events = $\frac{(Z\alpha/2+Z\beta)^2}{pq(logHR)^2}$

Probability of the event=1-($ps_{1\,(t)}$ +$qs_{2\,(t)}$)

Where n is the sample size; $Z\alpha$ /2 is a critical value of standardized normal distribution which is 1.96; β is power (80%), p is the proportion of population allocated for the first group proportion of the population allocated for the second group s1 (t) survival function in the first group at time t, s2 is survival function in the second group at time t and HR is the hazard ratio of each variable which are taken from similar previous studies. The freedman principle approach assumes equal allocation between these groups which means p=q=0.5 the largest required sample was 386. It can also be calculated by using STATA 14.1 software for survival analysis. STATA command 'power log-rank (cumulative probability of survival), HR (1.53) power (0.8) wd prob (0.1 **(15)** giving same sample size.

Sampling Technique and Sampling Procedure

All patients scheduled to undergo elective surgical procedures from September 1, 2017, to August 31, 2022, at Debre Markos Comprehensive Specialized Hospital were identified. The patient's medical card number was extracted from the scheduled cancellation registration book. There were a total of 1158 admitted elective surgical patients who experience at least one cancelation within four years. The 386 samples were proportionally allocated for each year and with systematic random sampling, the study participants of each year were selected as follows. First, numbering the unit of each year on the frame from 1 to N (N= total admission of each year), then we determine the sample interval (K) by dividing the number of units in the population by the desired sample size of each year (n=sample size of each year**) (Figure 2).**

Operational Definition

An adequate number of surgeons: is defined as assigned senior specialists in each operation suite and even if the team was not busy with emergency surgery **(16).**

Adequate number of nurses: is defined as assigned scrub and circulating nurse in each operation suite and even if the team was not busy with emergency surgery **(16)**

Adequate number of anesthetists: is defined as the presence of an assigned anesthetist in each operation suite and even if the team was not busy with emergency surgery **(16)**

Censored: patients who were referred to other hospitals after the first cancellation, died after the first cancellation and before surgery, against surgery after the first cancellation, and were absent for call to surgery.

Elective surgery: is non-emergency surgery that is medically necessary, but which can be delayed for at least 24 hours **(17).**

Event (failure): Elective surgery is done after the first cancellation.

First day of surgical cancellation: not being done with the first appointed day

Intended day: the specific and planned day when the operation is to be performed.

Major surgery: Surgery involving risk to the life of the patient specifically an operation on an organ

within the cranium, chest abdomen, and pelvic cavity **(18)**

Minor surgery: a set of procedures in which short surgical techniques are applied to superficial tissue, usually with local anesthesia **(18).**

Survival time: The time in days from the date of first cancellation to the date of the event or time of censoring.

Time to surgery: is defined as the time from the first day of cancellation of the elective case to the surgery done within days.

The Variables of the Study

Outcome variable

Time to surgery after the first cancellation

Independent Variables

Socio-demographic factors: age, sex, residency, occupation, religion, marital status, educational status, and employment status

Hospital administration-related factors: availability of recovery bed, consistency of power, availability of operation room supply including all surgical instrument set, presence of anesthesia drug supply, enough OR table, oxygen availability, and presence of cross-matched blood

Patient-related factors: patient acceptance, family or caregiver acceptance, other stable medical conditions, the patient being NPO, whether ordered laboratory investigation done on time, being a

member of health insurance schemes, and the responsible department (obstetrics, gynecology general surgery, and orthopedics)

Health professional-related factors: an adequate number of surgeons in all specialties, an adequate number of anesthetists, and an operating room nurse

Data Collection Procedures, Tools, and Data Quality Control

The patient's medical record numbers were retrieved from surgery cancellation logs. Data were collected using a structured checklist prepared in English. The data extraction sheet was adapted and modified from different related studies **(8, 19-24) (Figure 1)** and also from different registration books. Surgical intervention was confirmed by reviewing the operation note sheet from the patient chart in the hospital.

Data quality was assured by designing a proper data extraction checklist. Pretest was conducted on 5 %(19) of the sample size at DMCSH on charts of elective surgical patients who experienced at least one cancellation to check usually recorded variables on the patient s medical record and their consistency for the actual study. One day of training was given concerning the data extraction and the data collection process for data collectors. During the data collection time, close supervision and monitoring were carried out by the principal investigator to ensure the quality of the data; daily evaluation of the data for completeness and encountered difficulties at the time of data collection was attended to accordingly. Finally, all the collected data were checked for completeness and consistency during the data management, storage, and analysis.

Data Processing and Analysis

The collected data were checked for clarity, consistency, and completeness up to the end of the data collection period. After extraction, data were coded and entered into the Epidata version 3.1 statistical software and exported to STATA version 14.1(College Station, Texas 77845 USA) for cleaning recoding categorizing further analysis. The percentage, frequency, mean and median based on the distribution of each data about all covariates were summarized by descriptive statistics. The survival experience of the patients was assessed by using the Kaplan-Meier survival function. The log-rank test was used to test the presence of significant differences among survival curves.

The independent effects of predictor variables for surgery were assessed by Cox proportional hazard model. Schoenfeld residual tests, the interaction of each covariate with time, and graph methods were used to check the Cox proportional hazard (PH) assumption. The Cox PH model was done to estimate the independent effect of predictors on the occurrence of surgery after the first cancellation. Likely hood ratio (LR) was used to identify model fitness among the candidate's model and the model with high value considered well fit indicating less information lost on the data was selected and further goodness of fit of the final model was assessed by using Cox-snell residual technique. The model was built by a stepwise backward elimination procedure.

The potential predictors to the full model were selected by bi-variable Cox proportional hazard regression with the cut of point $P \leq 0.25$. The multi-collinearity for variables was checked using the variance inflation factor (mean VIF) and it was 1.12 indicating little or no collinearity between independent variables. The association between predictors and hazard of surgery was summarized using an Adjusted Hazard Ratio (AHR), and statistical significance was tested at $P<0.05$. The survival status and in-hospital surgery rate of the elective case after the first cancellation were estimated.

ETHICAL CONSIDERATION

The ethical principles were consistent with the Declaration of Helsinki. Ethical clearance was obtained from the Institutional Research Ethics Review Committee of Debremarkos University with reference number: HSC/R/C/Ser/PG/Co/50/11/14.The ethical letter was given to Debre Markos Comprehensive Specialized Hospital to get permission for the data collection process. A permission letter was obtained from Debre Markos Comprehensive Specialized Hospital. The necessary explanation regarding the purpose of the study was informed to data collectors and the concerned official bodies in the hospital. Confidentiality of the information was assured by not recording patients' names from the chart and privacy of the information was maintained. Informed written consent was not taken as secondary data were extracted from patient charts.

RESULTS

Sociodemographic and Other Characteristics

The analysis was performed for a total of 386 patients who met the inclusion criteria from September 1, 2017, to August 31, 2022. The mean age of the participants was 41.01$\pm$18.61 and with a range from 1 year to 85 years. The majority of the elective surgery cases, the one hundred and sixty-three (42.23%) cases after cancellation came from the surgical department, followed by one hundred and twenty-nine (33.42%), seventy-two (18.65) %) and twenty-two (5.7%) patients from the respective departments of Gynecology, Orthopedics and Obstetrics (**Table 1 and Table 4)**.

Table 1: Sociodemographic Characteristics of Elective Cases after First Cancellation in DMCSH Northwestern Ethiopia, September 1, 2017, to August 31, 2022

Sociodemographic Categories		Frequency	Percent (%)
Sex	Male	189	48.96
	Female	197	51.04
Religion	Orthodox	363	94.04
	Muslim	17	4.40
	Protestant	5	1.30
	Others	1	0.26
Marital Status	Single	95	24.61
	Married	264	68.39
	Divorced	11	2.85
	Widowed	8	2.07
	Separated	8	2.07
Residence	Urban	114	29.54
	Rural	272	70.46
Occupation	Employed	161	41.71
	Unemployed	225	58.29
Educational Status	Literate	107	27.72
	Illiterate	279	72.28

Table 2: Age Category of Elective Cases after First Cancellation in DMCSH, Northwestern Ethiopia from September 1, 2017, to August 31, 2022

Age Category	Frequency	Percent
1-5	14	3.6
6-10	3	.8
11-15	10	2.6
15-20	27	7
21-25	37	9.6
25-30	35	9.1
31-35	36	9.3
36-40	44	11.4
41-45	29	7.5
46-50	38	9.8
51-55	26	6.7
56-60	28	7.3
61-65	19	4.9
66-70	16	4.1
71-75	17	4.4
76-80	5	1.3
81-85	2	.5

Hospital Administration-Related Factors (Table 3)

Table 3: Hospital Administration Related Factor for Surgery after First Cancellation in DMCSH, Northwestern Ethiopia, September 1, 2017, to August 31, 2022

Variables	Categories	Frequency	Percent (%)
Presence of adequate	Yes	203	62.85
recovery bed	No	120	37.15
No interruption	Yes	267	82.66
of electrical power	No	56	17.34
Presence of essential	Yes	215	66.56
anesthesia drugs	No	108	33.44
Presence of cross	Yes	136	42.11
matched blood	No	187	57.89
Presence of oxygen	Yes	277	85.76
supply	No	46	14.24
Presence of gown	Yes	239	73.99
and drape	No	84	26.01
Presence of surgical	Yes	191	59.13
the instrument set	No	132	40.87
Presence of over	Yes	77	23.84
scheduling	No	246	76.16
Adequate number	yes	259	80.19
of OR table	No	64	19.81

Patient-Related Factor (Table 4)

Table 4: Patient-Related Factor for Surgery after First Cancellation in DMCSH, Northwestern Ethiopia, September 1, 2017, to August 31, 2022

Variables	Categories	Frequency	Percent (%
Being a member of	Yes	216	55.96
health insurance	No	170	44.04
Patient acceptance of	yes	337	87.31
about procedure	No	49	12.69
Family acceptance	Yes	330	85.49
of procedure	No	56	14.51
Patient being	Yes	306	79.27
NPO	No	80	20.73
Stable medical	Yes	279	72.28
condition	No	107	27.72
Lab investigation was done	Yes	224	58.03
	No	162	41.90
Department			
(Cases in each department)	General Surgery	163	42.23
	Gynecology	129	33.42
	Orthopedics	72	18.65
	Obstetrics	22	5.7

Health Professional Related Factor (Table 5)

Table 5: Health Professional-related Factor for Surgery after First Cancellation in DMCSH Northwestern, Ethiopia, September 1, 2017, to August 31, 2022

Variables	Categories	Frequency	Percent (%)
Adequate number of surgeon	Yes	277	71.76
	No	109	28.24
Adequate number of anesthetist	Yes	298	77.20
	No	88	22.80
Adequate number of nurse	Yes	357	92.49
	No	29	7.51

Survival Status and Survival Function of Elective Surgical Patients

The overall Kaplan-Meier estimate showed that the probability of surgery of elective case surgery was a long duration on the first day after the first cancellation and progressively short time as the follow-up time increased as shown in the figure below **(Figure 3).**

Survival Function and Comparison of Different Categories

Kaplan-Meier estimator survival curve gives the estimate of survivor function among different groups of variables to make comparisons. Separate graphs of the estimates of the Kaplan-Meier survivor functions were constructed for categorical variables as described below. In general, the pattern that one survivorship function lying lower than another means the group defined by the lower curve has a short time to elective surgery than the group defined by the upper curve or had a more favorable survival experience than the group defined by the upper. But the statistical

question is whether the observed difference seen on the plot is significant or not. This can be shown by the log-rank test.

The log-rank test was conducted to check for the existence of significant differences in survival among various levels of the categorical predictors considered in the study. The Kaplan –Meier analysis indicated significant evidence of differences in survival times in different categories among different predictor variables as shown below.

The Kaplan-Meier survival curves below showed those elective surgical patients after the first cancellation of urban residence had a short time of surgery than elective surgical cases after the first cancellation from rural residence **(Figure 4).**

The Kaplan-Meier survival curves below showed that elective surgical patients after the first cancellation with laboratory investigation had a short time to surgery than elective surgical patients from non-investigated **(Figure 5).**

The Kaplan-Meier survival curves below showed those elective surgical patients first cancellation being member of health insurance had a short time for surgery than elective surgical patients after the first cancellation of not being a member of health insurance **(Figure 6)**

The Kaplan-Meier survival curves below showed those elective surgical patients after first cancellation with stable medical conditions had a short time to surgery than elective surgical patients with no stable medical conditions **(Figure 7).**

Time to Surgery in Elective Surgical Patients after First Cancellation

Three hundred eighty-six study participants were followed in the study period, and the total person day of observation was 10013. The in-patient rate of surgery was 83.67% or 3 per 100 major surgery-day observations and 16.33% of elective cases were not done and the outcome of those in this study,50% was unknown, 26.65% were referred to other hospitals, 18.75% of cases were not operated with known unstable medical conditions and 7.81% died before the operation was performed. The median time to surgery was 25 days with an IQR of 17-40 days. The mean survival time for elective surgical patients after the first cancellation was 30.62 (95% CI 28.56-32.67) days.

Predictors of Time to Surgery for Elective Cases after First Cancellation

The Cox proportional hazard model showed that the sex, patient residence, educational status, occupational status, marital status, health insurance membership status, patient medical conditions, laboratory investigations, presence of cross-matched blood, availability of surgical instrument set, presence of oxygen and presence of assigned surgeon were candidate variables in the bivariable analysis with p-value $\leq$ 0.25. Then multivariate Cox regression model was conducted with selected eleven predictor variables that meet the assumption. The model was fitted with backward elimination variable selection technique and four variables become significant in the final multivariate Cox proportional hazard model. The significant variable (p-value of <0.05) in the final model were residence in the urban areas, laboratory investigation, being a member of health insurance, and stable medical conditions.

The multivariable Cox proportional hazard regression model shows that, at any given point in time, urban areas were 1.62 (AHR=1.62; 95% CI 1.26, 1.96) times more likely than rural areas to have a short time to surgery among elective surgical patients after the first cancellation, which was statistically significant. Among patients with elective surgery for whom there is a laboratory study, the probability of surgery at any given time point is almost 1.62 times (AHR = 1.62; 95% CI 1.29, 2.04) higher shortly before surgery than in patients without the study, by controlling the effect of other variables. The operation time at any point in elective surgery patients who were members of a health insurance scheme was 1.55 times shorter than that of elective surgery patients without health insurance (AHR=1.55, 95% CI 1.24, 1.96). Another statistically significant variable in this study was stable health status, which was 1.43 times (AHR=1.43; 95% CI 1.13, 1.79) higher than patients without stable health **(Table 6).**

Table 6: Bi-variable and Multivariable Cox Regression Analysis to Identify the Predictors of Surgery among Elective Cases after the First Cancellation at DMCSH, Northwestern Ethiopia, from September 1, 2017, to August 31, 2022

Variables	Categories	Survival status		CHR((95%CI)	AHR(95%CI)	P-value
		Event	Censored			
Sex (N=386)	Male	162	32	1	1	
	Female	161	31	0.71[0.56-0.88]	0.74[0.59-0.93]	P=0.11
Occupation (N=386)	Employed	144	27	1	1	
	Unemployed	189	36	0.70 [0.56-0.88]	0.76[0.60-0.96]	P=0.21
Residence (N=386)	Urban	83	17	1.58[1.22-2.04]	1.62[1.26-2.09]	P<0.001**
	Rural	240	46	1	1	
Educational status (N=386)	Literate	177	20	1.20[0.95-1.51]	1.19[0.95-1.50]	P=0.15
	Illiterate	206	43	1	1	
Presence of surgeon (N=386)	Yes	220	45	1	1	
	No	103	18	1.27[1.00-1.61]	1.33[1.05-1.70]	P=0.18
Member of Health insurance (N=386)	Yes	172	39	1.48[1.18-1.85]	1.55[1.24-1.96]	P<0.001**
	No	151	24	1	1	
Medical Condition	Yes	195	40	1.39[1.11-1.74]	1.43[1.13-1.77]	P=0.002**
	No	128	23	1	1	
Whether ordered laboratory investigations were done (N=386)	Yes	189	28	1.44[1.15-1.80]	1.62[1.29-2.04]	P<0.001**
	No	134	35	1	0.83[0.66-1.05]	
Surgical Instrument set (N=386)	Yes	191	52	1	1	
	No	132	11	1.34[1.07-1.67]	1.18[0.94-1.49]	P=0.14
Availability of Oxygen (N=386)	Yes	269	58	1	1	
	No	54	5	1.21[0.90-1.63]	1.06[0.78-1.43]	P=0.68
Cross-matched blood (N=386)	Yes	136	24	1	1	
	No	187	39	0.85[0.68-1.06]	0.86[0.68-1.09]	P=0.22
Recovery bed availability	Yes	213	50	1	1	
	No	110	13	1.17[0.93-1.48]	1.12[0.89-1.42]	P=0.30

** Indicated significant predictors

Assessing the Fitness of the Final Model

Model fitness was assessed using the Cox-snail residual test. The cox-snail residual test showed that the hazard function follows closely the 45-degree line except for a large value of time and it is common for models with censored data to have some wiggling at a large value of time which shouldn't be much concern. This was understood that it had approximately an exponential distribution with a hazard rate of one and that the model fits the data well **(Figure 8).**

DISCUSSION

This study provided the first evidence of predictive factors for time to surgery after first cancellation among elective surgery cases admitted to Debremarkos Comprehensive Specialized Hospital. There were 323 (83.6%) [95% CI: 79.6, 87.05] surgeries, or 3 out of 100 major surgeries-day observation after the first cancellation. Residence in an urban area, the status of ordered laboratory tests, health insurance membership, and stable medical condition were considered important predictors of time to surgery after the first cancellation.

In terms of rates of surgery after first cancellation, this is comparable to studies done in Addis Ababa, specifically studies conducted at Zewditu Memorial Hospital in Ethiopia (86% [16], compared with studies done in the UK at 80% [22]), The study conducted in Iran hospital of Social Security Organization was 87% **[21]**. The study conducted in Uganda, especially at Mulago Hospital was 80% **(1).**The mean survival time was 30.62 days [95% CI: 28.56; 32.67), longer than the time outlined in FMOH (Federal Department of Health) guidelines for saving lives through safe surgical care and anesthesia (SALT) with a waiting time of 14.5 days **(13).**

On the other hand, the surgery rate of elective cases after the first cancellation indicated in the present study is lower than the studies reported from Canada 92% **(20)**, the studies in Burkina Faso (89%) **(25)**, the studies Mahatma Gandhi medical college, Ethiopia (95%) **(26).** A higher level of elective surgery in developed countries may be related to the quality of operating theaters, sufficient staff training, level of public awareness, up-to-date equipment, and high availability of services. However, the rate of surgery after the first cancellation obtained from this study is higher than the study conducted in Ghana, specifically Comfy Anokye teaching hospital (50%) **(27),** Zambia (77%) **(28),** and study in Tikur Anbesa Specialized Hospital, Ethiopia (70.8) **(29).** The

discrepancy may be due to differences in study duration, study population, and duration of some studies, another reason may be low patient flow in the study setting. In this study, the urban residence was found to be an important predictor of surgery done after experiencing first cancellation among elective surgical patients compared to rural areas. This finding is in agreement with other previous studies conducted in Portuguese **(15),** study in Tanzania **(30),** study in a specialized hospital in Ethiopia **(17).**

For those elective surgical cases after the first cancellation, ordered laboratory investigation being done was observed as an independent predictor of surgery after the first cancellation, when compared to patients not done as ordered. This is in agreement with previous studies in Wales **(31),** and a study in Nigeria **(32).** Despite sending general laboratory tests for each scheduled surgical case before surgery, the study confirmed that it was not completed on time as directed due to long waiting times.

In addition, health insurance coverage was another predictor of scheduled intraoperative surgery after initial cancellation compared to those without health insurance. This finding is supported by different studies conducted in Australia **(33),** and the study in Nigeria **(32).** These people were more likely to have surgery after their first cancellation because they paid per family per year at a lower or lower rate, making them more likely to go to public hospitals out of pocket for each visit. **[26,28].**

Moreover, the stable state of health before elective surgery is another predictor of surgery, and among the elective cases after the first cancellation, patients with stable health before surgery were an important predictor. This study is in line with the study conducted by Hawasa Comprehensive Specialized Hospital, Ethiopia **(34),** and with a study conducted in Portuguese **(15).** A stable medical condition is a major concern before elective surgery, as it causes difficulties in anesthesia induction and leads to re-cancellation delaying the actual surgical time.

The retrospective nature of the present study and the incomplete history of patients undergoing elective surgery were limitations of the study. The problem of missing data can lead to random error and patients with missing data are systematically different from patients with complete data. Additionally, some patients may be denied phone calls and some surgeries may be performed at other nearby hospitals. Loss of follow-up may have affected the rate of hospital surgery.

CONCLUSION AND RECOMMENDATION

The in-patient surgery rate after the first cancellation was 83.6% despite many patients requiring surgery, but comparable to many previous findings. The total person-days of observation were 10013 or 3 out of 100 elective major surgeries per day observation after the first cancellation. The median survival time was 25 (IQR:17-40 days). The mean survival time for elective surgery after the first cancellation was 30.62 (95% CI:28.56;32.67) days. In this study, urban residence, health insurance coverage, scheduled laboratory tests, and stable health status at the time of surgical admission were significant predictors of the time to elective hospital surgery after the first cancellation.

Our recommendations include rigorous follow-up of patients, along with timely completion of investigations, encouraging them to become members of health insurance schemes, raising awareness of its benefits, and monitoring other comorbidities. Researchers and the scientific community are encouraged to conduct multicenter follow-up studies with strong study designs with large sample sizes that can approximate the burden of this underestimated public health problem to estimate rates of elective surgery after initial cancellation. This will help to include some omitted variables for analysis in the current study that may be related to schedule activity prediction.

ABBREVIATIONS AND ACRONYMS

AHR: Adjusted hazard ratio, CC: Case cancellation, DMCSH: Debremarkos Comprehensive Specialized Hospital, DMU: Debre Markos University, DOS: Day of Surgery, DOSA: Day of Surgery Admission, FMOH: Federal Ministry of Health, NPO: Nothing Per OS, OR: Operation Room, ORT: Operation Room Theatre, PH: Proportional Hazard, SALTS saving lives through safe surgery and anesthesia, TASH: Tikur Anbesa Specialized Hospital, SD: Standard deviation, VIF: Variance inflation Factor

CONSENT FOR PUBLICATION

Not applicable

AVAILABILITY OF DATA AND MATERIALS

Available from the main author upon reasonable request

CONFLICT OF INTERESTS

The authors declare that they have no competing interests.

FUNDING

The study was not funded by any agent

AUTHORS' CONTRIBUTIONS

YA conceived and designed the study, performed analysis, interpreted the data, and prepared and drafted the manuscript.**ZA, LY, MM, GD, and MS** were involved in the design, conception, analysis, and interpretation of data and in drafting the manuscript. All authors read and approved the final manuscript.

ACKNOWLEDGMENTS

We would like to express our sincere appreciation to Dube Jara for his expert advice and supportive nature. Additionally, we are highly indebted to data collectors, and supervisors.

REFERENCES

1. Kikomeko S, Muwonge H, Ankarali H, Sserwanja Q, Timarwa AE. Waiting-time to Elective Surgery amongst Patients Attending Mulago National Referral Hospital (Uganda): A Cross-Sectional Study. 2020.

2. Diaz A, Sarac BA, Schoenbrunner AR, Janis JE, Pawlik TM. Elective surgery in the time of COVID-19. The American Journal of Surgery. 2020;219(6):900-2.

3. Encyclopedia. Elective surgery. 2021.

4. Bari S, Incorvia J, Iverson KR, Bekele A, Garringer K, Ahearn O, et al. Surgical data strengthening in Ethiopia: results of a Kirkpatrick framework evaluation of a data quality intervention. GLOBAL HEALTH. 2021;14:1855808.

5. Abásolo I, Barber P, López-Valcárcel BG, Jiménez O. Real waiting times for surgery. Proposal for an improved system for their management. Gac Sanit. 2014;28(3).

6. Okeke C, Obi A, Tijani K, Eni U, Okorie C. Cancellation of elective surgical cases in a Nigerian teaching hospital. Frequency and reasons. Niger J Clin Pract. 2020;23(7):965.

7. Yohanes YB, Sinshaw AE, Ayisa AA. Root causes of elective surgical case cancellation in Ethiopia: a systematic review and meta-analysis. 2020.

8. Siciliani L, Hurst J. Explaining waiting for times variations for elective surgery across OECD countries. 2003.

9. Hori Y, Nakayama A, Sakamoto A. Surgery cancellations after entering the operating room. Surgery. 2016;4(2):6.7.

10. Gebresellassie HW, Tamerat G. Audit of surgical services in a teaching hospital in Addis Ababa, Ethiopia. clinic. 2019;5802:54.3.

11. McIntyre D, Chow CK. Waiting time as an indicator for health services under strain: a narrative review. INQUIRY: The Journal of Health Care Organization, Provision, and Financing. 2020;57:0046958020910305.

12. Organization WH. Surgical care systems strengthening: developing national surgical, obstetric, and anesthesia plans. 2017.

13. Ethiopia Fmoho. Saving life through safe surgery and anesthesia strategic plan. 2016-2020.

14. Hospital dM. elective surgery and cancellation. 2021.

15.De Cima JDF. Econometric evaluation of waiting times for scheduled surgery in the Portuguese NHS. 2021.

16. Mesele E. POLICY ANALYSIS ON ACCESSIBILITY OF ESSENTIAL AND EMERGENCY SURGICAL AND ANESTHESIA CARE IN DEVELOPING COUNTRIES: THE CASE OF ETHIOPIA.

17. Ayele A, Weldeyohannes M, Tekalegn Y. Magnitude and reasons for surgical case cancellation at a specialized Hospital in Ethiopia. J Anesth Clin Res. 2019;10(927):2.

18. Okeke C, Obi A, Tijani K, Eni U, Okorie C. Cancellation of Elective Surgical Cases in a Nigerian Teaching Hospital: Frequency and Reasons. Nigerian Journal of Clinical Practice. 2020;23(7).

19. Stahlschmidt A, Novelo B, Freitas LA, Passos SC, Dussán-Sarria JA, Félix EA, et al. Predictors of in-hospital mortality in patients undergoing elective surgery in a university hospital: a prospective cohort. Revista brasileira de anestesiologia. 2018;68:492-8.

20. Sommer JL, Jacobsohn E, El-Gabalawy R. Impacts of elective surgical cancellations and postponements in Canada. Canadian Journal of Anesthesia/Journal canadien d'anesthésie. 2021;68(3):315-23.

21. Ray S, Kirtania J. Waiting time of inpatients before elective surgical procedures at a State Government Teaching Hospital in India. Indian Journal of public health. 2017;61(4):284.

22. Oudhoff J, Timmermans D, Knol D, Bijnen A, Van der Wal G. Waiting for elective general surgery: impact on health-related quality of life and psychosocial consequences. BMC Public Health. 2007;7(1):1-10.

23. Bagilkar VV, Lamba D, Mehertab M. To assess the rationale of cancellation of surgical patients for elective surgery and length of hospital stay at Jimma University Medical Centre, Oromia Region, Ethiopia. Medical Science. 2020;24(106):4813-20.

24. Ogwal A, Oyania F, Nkonge E, Makumbi T, Galukande M. Prevalence and Predictors of Cancellation of Elective Surgical Procedures at a Tertiary Hospital in Uganda: A Cross-Sectional Study. Surgery Research and Practice. 2020:NA.

25. Lankoande M, Bonkoungou P, Traore S, Kabore R, Ouangre E, Pendeville P. Cancellation of elective surgical procedures in the university teaching hospital center Yalgado Ouedraogo in Burkina Faso: incidence, reasons, and proposals for improvement. Southern African Journal of Anaesthesia & Analgesia. 2016;22(5).

26. Nanjappa B, Kabeer KK, Smile SR. ELECTIVE SURGICAL CASE CANCELLATION-AN AUDIT. International Journal of Current Research and Review. 2014;6(24):19.

27. Anarfi N, Singh S, Nakua E. Effect of Cancellation of Elective Operation on Patient. IAR Journal of Anaesthesiology and Critical Care. 2020;1(1).

28. Musonda M, Choolwe J, Jean R, Jakub G, Chiara P, Cheelo M. Factors Associated with Waiting Time for Patients Scheduled for Elective Surgical Procedures at the University Teaching Hospital (UTH) in Zambia. Annals of Medical and Health Sciences Research. 2020.

29. Genetu A, Ademe Y, Leake T, Aderaw H, Bekele A. OPERATING ROOM EFFICIENCY IN A TERTIARY CENTER IN ETHIOPIA.

30. Chalya P, Gilyoma J, Mabula J, Simbila S, Ngayomela I, Chandika A, et al. Incidence, causes, and pattern of cancellation of Elective surgical operations in a University Teaching Hospital in the Lake Zone, Tanzania. African Health Sciences. 2011;11(3):438-43.

31. Oudhoff JP, Timmermans DR, Rietberg M, Knol DL, Van Der Wal G. The acceptability of waiting times for elective general surgery and the appropriateness of prioritizing patients. BMC Health Services Research. 2007;7(1):1-12.

32. Nnabugwu II, Nwankwor C, Ugwumba FO. Gaining Access to Major Elective Surgeries in a Public Tertiary Health Institution in Southeast Nigeria: Evaluating Household Payment Coping Strategies. ANNALS of AFRICAN SURGERY. 2019;16(2):1.

33. Clover KA, Dobbins TA, Sanson- Fisher RW, Smyth TJ. Factors associated with waiting time for surgery. Medical Journal of Australia. 1998;169(9):464-8.

34. Desta M, Manaye A, Tefera A, Worku A, Wale A, Mebrat A, et al. Incidence and causes of cancellations of elective operation on the intended day of surgery at a tertiary referral academic medical center in Ethiopia. 2018.

Click here to access/download;Figure;Figure 1.docx

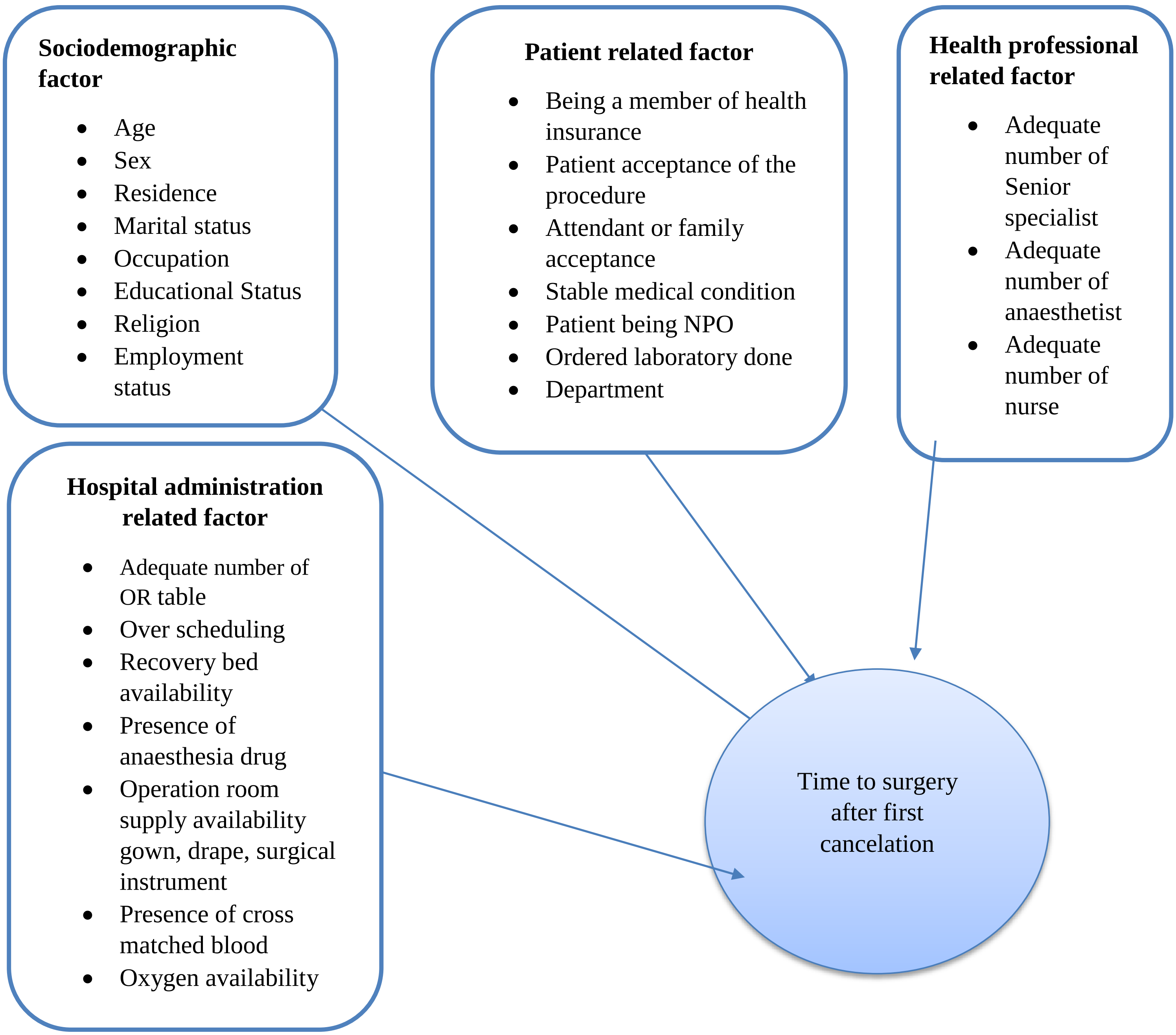

Figure 1: Conceptual Framework of Different Variables Adapted from Different Literature among Elective Cases after First Cancellation in Debre markos Comprehensive Specialized Hospital, Northwestern Ethiopia, from September 1/2017 to August 31/2022

Click here to access/download;Figure;Figure 2.docx

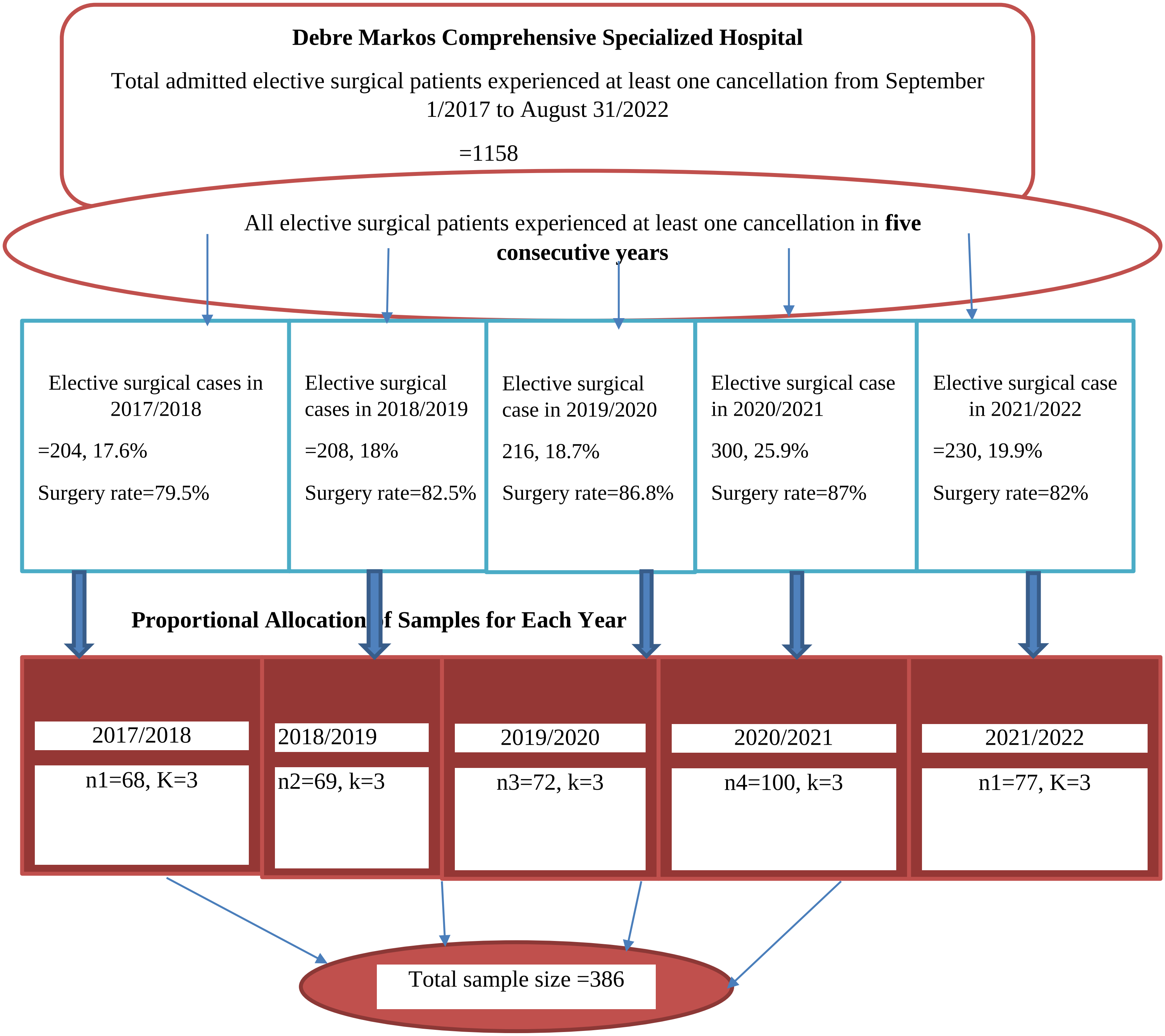

Figure 1: Schematic Presentation of Sampling Procedure to Elective Surgery Cases after First Cancellation in DMCSH, Northwestern Ethiopia from September 1/2017 to August 31/2022

Click here to access/download;Figure;Figure 3.docx

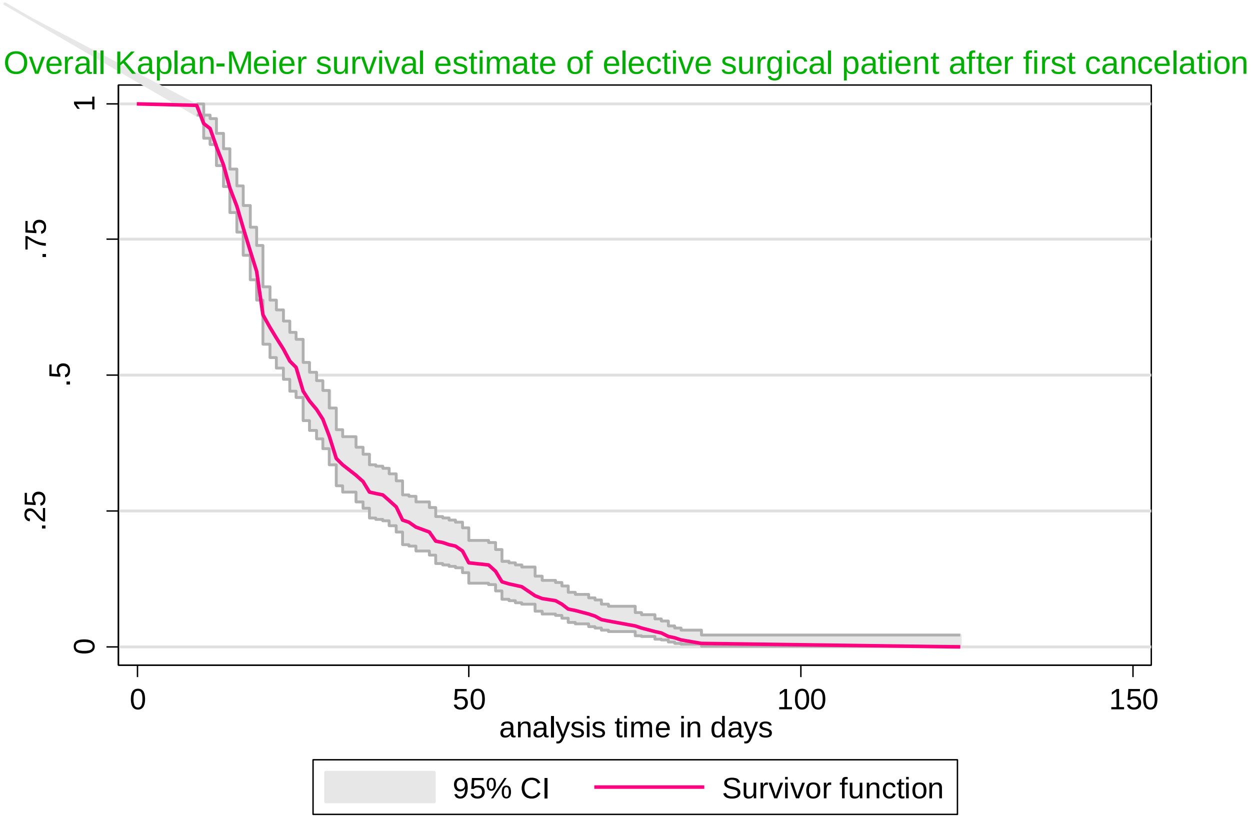

Figure 3: Overall Kaplan-Meier Survival Estimate of Elective Surgery Cases after First Cancellation in DMCSH, Northwestern Ethiopia from September 1/2017 to August 31/2022

Click here to access/download;Figure;Figure 4.docx

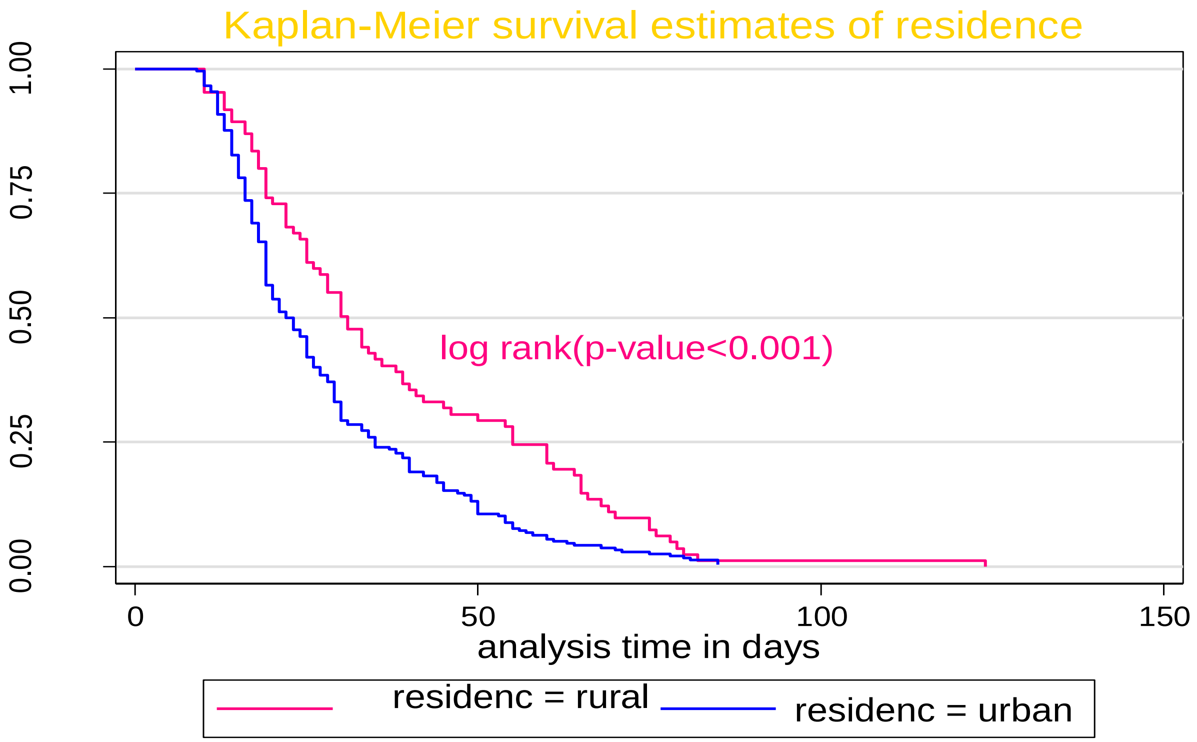

Figure 4: The Kaplan-Meier Survival Curves Comparing Survival Time of Elective Surgery Cases **of First Cancellation with Categories of Residence in DMCSH, Northwestern Ethiopia** from September 1/2017 to August 31/2022

Click here to access/download;Figure;Figure 5.docx

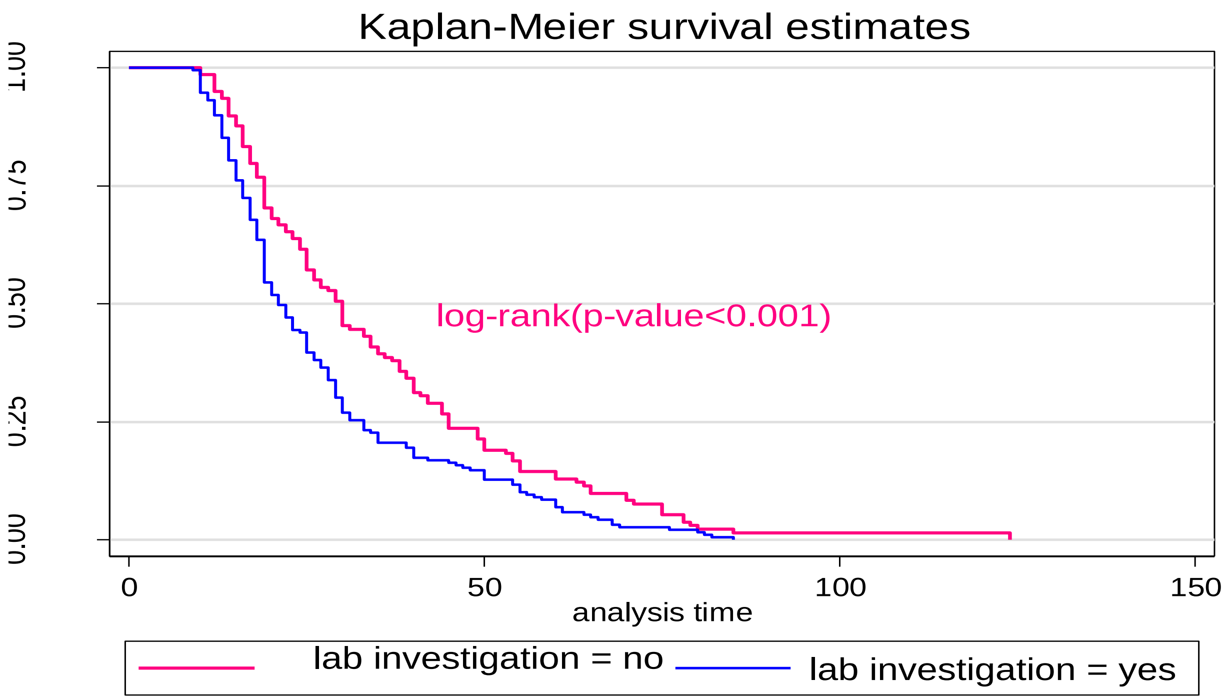

Figure 1: The Kaplan-Meier survival Curves Comparing Survival Time of Elective Surgery Cases after First Cancellation with Status of Laboratory Investigations in DMCSH, Northwestern Ethiopia from September 1/2017 to August 31/2022

Click here to access/download;Figure;Figure 6.docx

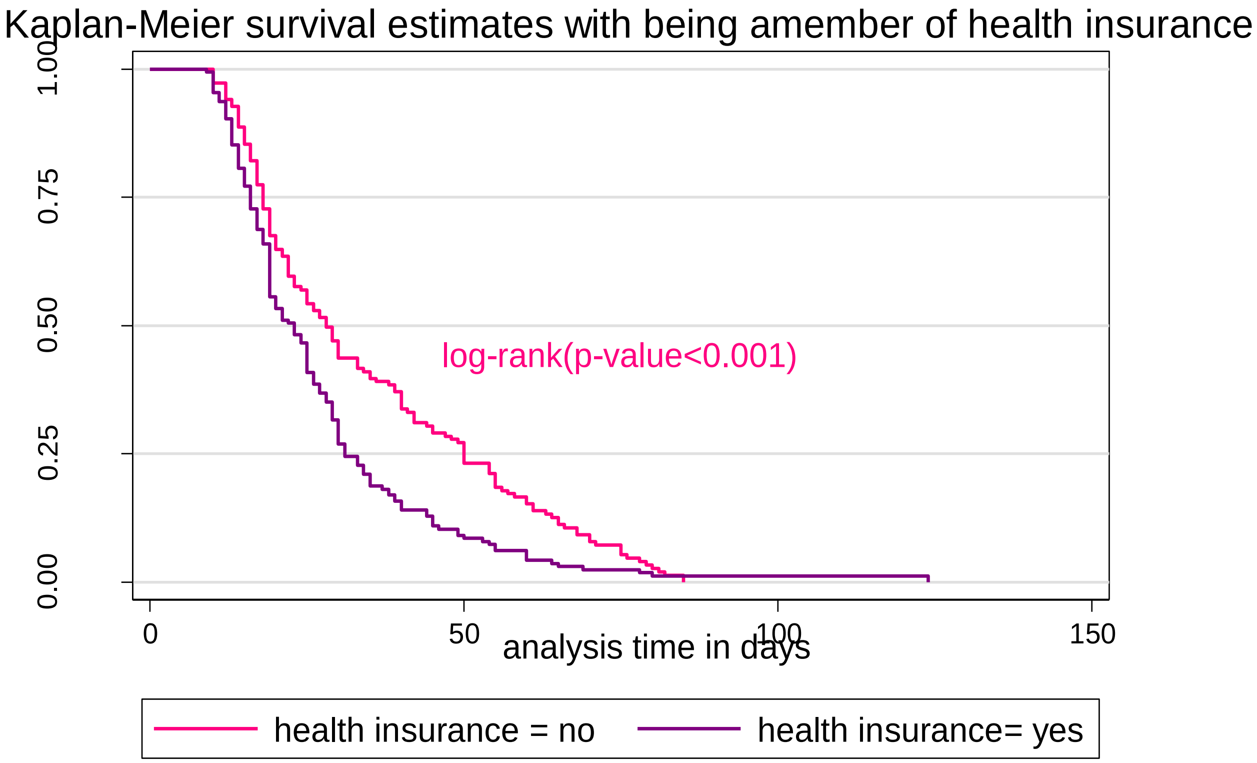

Figure 1: The Kaplan-Meier Survival Curves with Log-rank Test Comparing Survival Time of Elective Surgery Cases after First Cancellation with Categories of Membership of Health Insurance in DMCSH, Northwestern Ethiopia from September 1/2017 to August 31/2022

Click here to access/download;Figure;Figure 7.docx

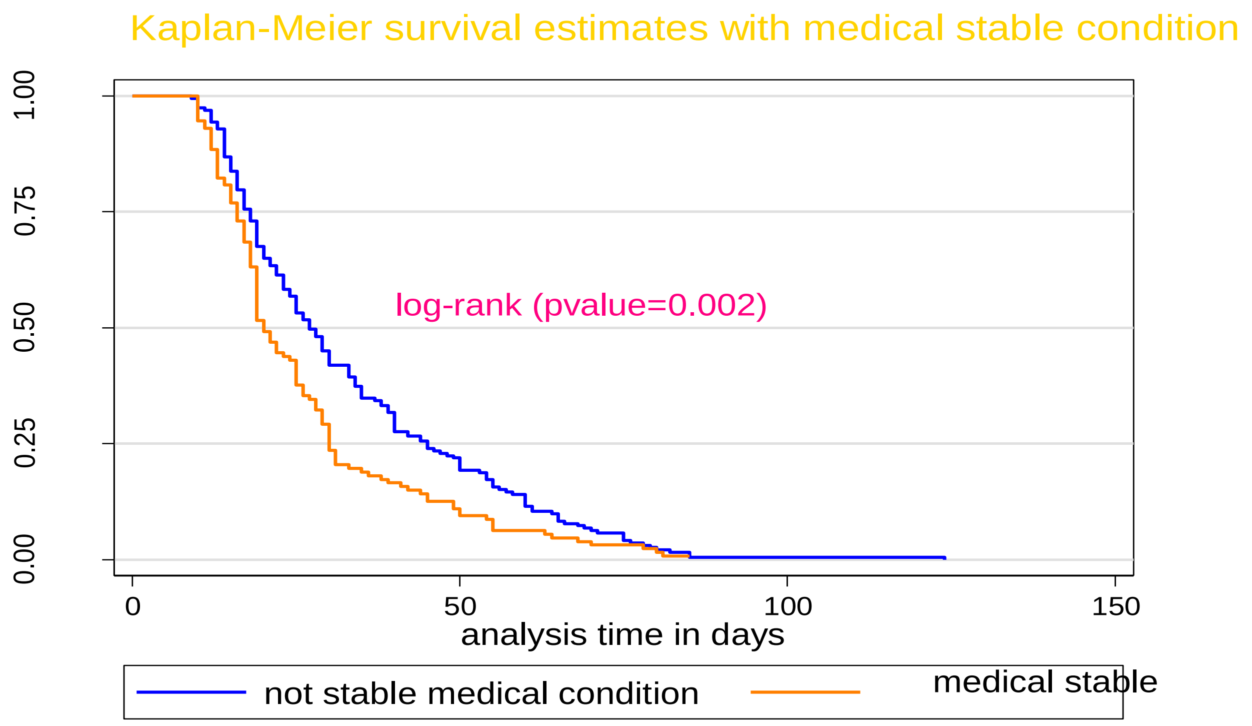

Figure 7: The Kaplan-Meier Survival Curves with Log-rank Test comparing survival Time of Elective Surgery Cases after First Cancellation with Categories of Medical Condition in DMCSH, Northwestern Ethiopia from September 1/2017 to August 31/2022

Click here to access/download;Figure;Figure 8.docx

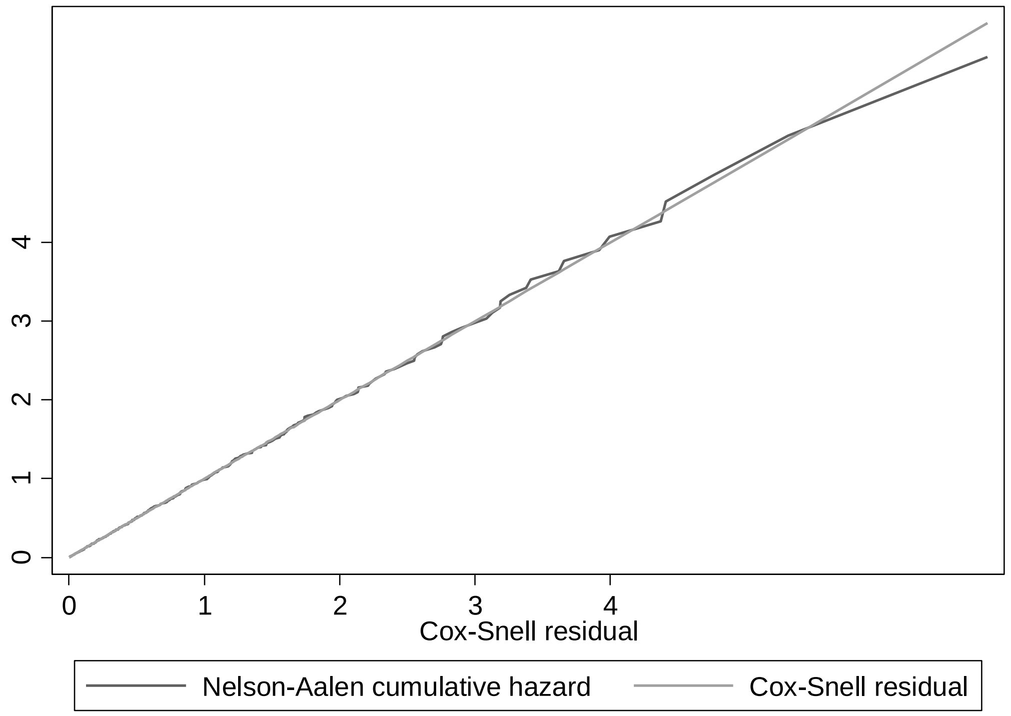

Figure 1: Assessment of Goodness of Model Fitness by Using Cox-Snail Residual Test for Time to Surgery and Its Predictors among Elective Cases Admitted at DMCSH, Northwestern Ethiopia from September 1/2017 to August 31/2022
