# Supplementary material for: Time to elective surgery and its predictors after first cancellation at Debremarkos Comprehensive Specialized Hospital, Northwest Ethiopia
Source: PLOS Glob Public Health. 2024 Jan 17;4(1):e0002183. doi: 10.1371/journal.pgph.0002183 (PMC10793890; doi:10.1371/journal.pgph.0002183)
Supplement: Attachment — Submitted filename: PLOS Global Public Health_DFB.docx [file pgph.0002183.s001.docx]

**PLOS Global Public Health**

**Time to Surgery after First Cancellation and Its Predictors in Elective Surgery Cases at**

**Debre Markos Comprehensive Specialized Hospital**

I read the manuscript with interest. It addresses a very important issue: the timing of surgery following the first cancellation and its predictors in elective procedures. These factors can have a significant impact on patient outcomes and healthcare system efficiency. By identifying and addressing potential predictors, we can optimize scheduling, reduce patient anxiety, enhance patient satisfaction, and improve overall surgical care delivery.

I herewith forward my input

**Abstract**

1. The abstract result section lacks some more data rather they gave detail conclusion content some of which are outside their finding.

**Introduction**

2. On page 3, the way the text is written looks like result or discussion section.
3. ‘’ *Our observations showed that many patients suffer from severe pain, additional costs, emotional trauma, and feelings of hopelessness, and eventually, the patient may die if the operation is canceled again on the scheduled date.’’*
4. On same page just after the above paragraph, the sentence lacks clarity and it would be good to rewrite as
   a. ‘’ A better assessment and understanding of factors that affect time to surgery after a first due date cancellation can help to reduce complications, re-operations, and unnecessary hospital stays. In turn, this can improve quality of care for patients who have undergone elective surgery.’’
5. *~~in~~ at Debre Markos Comprehensive Specialized Hospital, Northwestern Ethiopia.*
6. The following sentence need rewriting.
   b. *This study will help clinicians and other service providers design interventions to reduce surgical time in hospitals by removing predictable factors that could be avoided.\*

**Method**

7. Sample size calculation (Fig 1): how they were sure to achieve the target sample size and did not have contingency?
8. It is good authors made sampling proportionally allocated for each year. However, why they could not do that for each department (General Surgery, Gynecology, Obstetrics, and Orthopedics)? As the authors had not make proportional allocation for the departments, how did they address such confounding by indication factors? Another sticking issue, how did they deal with Gyn/Obs cases? What if a scheduled CS delivery cancelled for obvious reasons (initially schedule could because of risk to the mother and latter risk could be ruled out).
9. Operational definition: This section is not should be placed separately. These terms should be explained under each variable. E.g state the outcome variable and define it, the same should apply for other operation definitions to be placed under each respective.
   a. Authors can also those definitions as supplement file (if need be).
10. Overall, the method lacks clarity how data was collectors for non-patient related factors. How could it be possible to get such detail information for surgery that was cancelled 4-5 years ago? (For instance, adequate OR table (Y/N: where did they get this information? I am not sure if this data is documented on patient chart. Table 5 data looks unrealistic all add up 386 (was this too from patient chart?)
11. Table 6 also indicates no missing case at all. How did the authors achieve this precision? They might have selected patient cases only with 100% complete data with risk of selection bias.

Result

12. In all tables put Frequency and % in one column as N (%)
13. For marital status I would suggest making it Married /Single regardless of the reason for being single. That give good number for analysis and interpretation.
14. Put Table 1 and 2 as Table 1, and important to regroup logically (eg biological children adult and geriatric).
15. I would suggest putting Table 3, 4 and 5 as one Table 2 segregated by the three factors.
16. Refer my #10 feedback on method section.
