# Supplementary material for: Time to elective surgery and its predictors after first cancellation at Debremarkos Comprehensive Specialized Hospital, Northwest Ethiopia
Source: PLOS Glob Public Health. 2024 Jan 17;4(1):e0002183. doi: 10.1371/journal.pgph.0002183 (PMC10793890; doi:10.1371/journal.pgph.0002183)
Supplement: Attachment — Submitted filename: Response for Editors and Reviewers (Yib).docx [file pgph.0002183.s003.docx]

**Dear Editors and Reviewers in PLOS Global Public Health**

I would like to thank you for providing constructive comments and for devoting time to review the paper with manuscript number, **‘PGPH-D-23-00985’**, entitled as **‘Time to Elective Surgery and Its Predictors after First Cancellation at Debremarkos Comprehensive Specialized Hospital, Northwest Ethiopia’**. Most comments or corrections have been incorporated with clear descriptions in the paper, and some responses to editors and reviewers have been presented below.

1. In the sample size calculation, the rate of contingency was expressed as proportion to withdraw as 10% or 0.1
2. Sampling proportion was allocated for each year because the lists of elective surgery patients after cancellation were easily accessed from Surgery cancellation logbook in each year. Cases in each department were discovered mixed up or in disorganized circumstances in a single cancellation log book. The hospital has had a single OR shared by obstetrics, gynecology, general surgery, and orthopedics departments. It was preferable to not include cesarean delivery cases in obstetrics so that other cases could have equivalent time estimates to surgery. We decided to consider them as comparable study populations because of the sole common OR. They were thought to have comparable surgical probabilities because elements including the extent of their underlying medical conditions, the results of diagnostic or laboratory testing, and other hospital-related issues were assumed to be common across all departments. However, once they were canceled, the majority of obstetric cases were handled in a week, which is taken as one of drawbacks of the study.
3. Some institutional resource-related factors (non-patient related) like adequate OR table, anesthetists or nurses were rectified as **availability of each professional in each OR**. The data were collected both from cancellation logbook and patient charts. For all patients canceled from surgery, the reason of cancellation was clearly documented in a separate cancellation logbook as the respective surgeon was not available, lack of OR material, lack of blood, lack of assigned nurses, interruption of electric supply/power supply. So, in the paper, it has been tried to make it objective and measurable.
4. The total person-times of observation were 10013 person-days. These Person-days represent a rough estimate of the total number of time-at-risk days that all study participants contributed.
5. Minor surgeries were excluded because the procedures were performed in a separate minor OR, which had totally different factors affecting it as compared to the major surgeries.
6. The sample size was calculated for both objectives. The final sample size which was larger was taken from the second objective ( using AHR=1.53, probability of event 0.5, event 174, proportion to withdraw or incomplete charts (10%), and other assumptions mentioned in the paper).
7. Systematic sampling was easier and more direct in terms of sample technique. It might also make it easier to finish a relatively long study period of time. It required less time and resources to implement and could cover a large population.
8. Our study had a right censored data; example, patients who were waiting the appointment at the end of the follow-up period.
9. The primary objective was to evaluate the time to surgery interms of median survival time or mean survival time. In time to event analysis, both median and mean time to event can be understood from Kaplan-meier survival curve. If we say ‘time to surgery’, it literally describes any amount of time irrespective of the model or consideration of censored data. So, we are obliged to say ‘survival time to surgery’.
10. The majority of important data were included in the manuscript. The rest will be available upon reasonable request for readers.
11. **The figure** formats (.tiff format) with appropriate sizes have been attached.
