# Supplementary material for: Time to elective surgery and its predictors after first cancellation at Debremarkos Comprehensive Specialized Hospital, Northwest Ethiopia
Source: PLOS Glob Public Health. 2024 Jan 17;4(1):e0002183. doi: 10.1371/journal.pgph.0002183 (PMC10793890; doi:10.1371/journal.pgph.0002183)
Supplement: Attachment — Submitted filename: PLOS Global Public Health_DFB_R2.docx [file pgph.0002183.s004.docx]

**PLOS Global Public Health**

**Time to Surgery after First Cancellation and Its Predictors in Elective Surgery Cases at**

**Debre Markos Comprehensive Specialized Hospital**

Thank you for authors in addressing my feedback. Overall, methodological feedback is not addressed well. Authors should add more information on method section and/or address the limitations on discussion section. Here is my re-revision embedded on my pervious comment.

**Abstract**

1. On method and result section I can see data on sex and other socio demographic variables. It is good to have such basic data on the abstract.
2. On conclusion part, I would remove this sentence as it is already presented in the result section and the subsequent sentence repeat the idea.

*~~‘’The time to surgery wa shortened by community health insurance coverage, urban residence, a stable other medical condition, and timely completion of diagnostic or laboratory tests.’~~*~~’~~

3. I would rewrite this sentence too as there may other variables clinician should focus though not statically significant on this study or authors did not collect on other important variables*. ‘’ Clinicians must focus on timely ordering and completion of diagnostic or laboratory tests, facilitating health insurance coverage, and comprehensive assessment and treatment of any coexisting medical conditions.’’*

**Introduction**

- My feedback well addressed.

**Method**

4. It is good authors redefine their variables; however, it is not still clear where did they get those data. Was it possible to get a five-year data on variables such as hospital and healthcare workers related?

My previous input: Overall, the method lacks clarity how data was collectors for non-patient related factors. How could it be possible to get such detail information for surgery that was cancelled 4-5 years ago? (For instance, adequate OR table (Y/N: where did they get this information? I am not sure if this data is documented on patient chart. Table 5 data looks unrealistic all add up 386 (was this too from patient chart?)

5. Authors still did not give justification for my previous input

Table 6 also indicates no missing case at all. How did the authors achieve this precision? They might have selected patient cases only with 100% complete data with risk of selection bias.

Result

6. My feedback addressed though previous Table 6 (current table 1-3), sample size add up 386. Practically impossible to have data without missing. Authors should justify this (did they include cases only with complete information?).

Discussion

7. I would not claim this is the 1^st^ study in Ethiopia, Check out t <https://bmcresnotes.biomedcentral.com/articles/10.1186/s13104-019-4709-y>
