# Supplementary material for: Time to elective surgery and its predictors after first cancellation at Debremarkos Comprehensive Specialized Hospital, Northwest Ethiopia
Source: PLOS Glob Public Health. 2024 Jan 17;4(1):e0002183. doi: 10.1371/journal.pgph.0002183 (PMC10793890; doi:10.1371/journal.pgph.0002183)
Supplement: Attachment — Submitted filename: Response for Editors and Reviewers.docx [file pgph.0002183.s005.docx]

**Dear Editors and Reviewers in PLOS Global Public Health**

I appreciate you for your constructive feedback and taking the time to read the paper with the manuscript number, **‘**PGPH-D-23-00985R1’, entitled as **‘Time to Elective Surgery and Its Predictors after First Cancellation at Debremarkos Comprehensive Specialized Hospital, Northwest Ethiopia’**. The majority of the reviewers' and editors' comments were handled in the previous revision, and both reviewers agreed that the comments were addressed. Some recent revisions and responses to editors and reviewers are provided here, and adjustments have been included with detailed descriptions in the text.

1. A list of all the factors that are routinely recorded in the Debremarkos Comprehensive Specialized Hospital surgery cancellation logbook, including hospital and healthcare worker-related factors like an adequate operating room table, anesthetists or nurses were corrected as availability of each material or professional in each OR. The data were collected both from cancellation logbook and patient charts. For all patients canceled from surgery, the reason of cancellation was clearly documented in a separate cancellation logbook as the respective surgeon was not available, lack of OR material, lack of blood, lack of assigned nurses, interruption of electric supply/power supply. So, in the paper, it has been tried to make it objective and measurable. It was possible to evaluate the following factors spanning five years **from both patient charts and cancellation log books.**
A. The availability of a recovery bed
B. No interruption of the electrical supply
C. Essential anesthesia drugs were present
D. Cross-matched blood was available
E. Accessibility of an oxygen supply
F. Gown and drapes were present
G. A complete set of surgical instruments was present
H. The schedule was too much
I. An operating table was available
J. Enrolled in community health insurance
K. Acceptance of the operation by the patient
L. The family's agreement to the operation
M. The patient remained NPO
N. Stable other medical conditions,
O. Diagnostic or laboratory tests completed timely
P. The assigned surgeon was available
Q. Availability of assigned anesthetist
R. The presence of an operating room nurse
2. The 2^nd^ concern raised was on 100% precision meaning without missing data. This might most likely be explained by the fact that data collectors only included cases with 100% complete data that demonstrated selection bias. Thus, one of the study’s drawbacks has been identified as selection bias.
